# *Lactiplantibacillus plantarum* OLL2712 Induces Autophagy via MYD88 and Strengthens Tight Junction Integrity to Promote the Barrier Function in Intestinal Epithelial Cells

**DOI:** 10.3390/nu15122655

**Published:** 2023-06-07

**Authors:** Yumiko Watanabe-Yasuoka, Ayako Gotou, Shigeomi Shimizu, Toshihiro Sashihara

**Affiliations:** 1 Food Microbiology and Function Research Laboratories, Division of Research and Development, Meiji Co., Ltd., Hachiouji, Tokyo 192-0919, Japan; ayako.gotou@meiji.com (A.G.); toshihiro.sashihara@meiji.com (T.S.); 2 Department of Pathological Cell Biology, Medical Research Institute, Tokyo Medical and Dental University, Tokyo 113-8510, Japan; shimizu.pcb@mri.tmd.ac.jp

**Keywords:** lactic acid bacteria, autophagy, intestinal barrier, mucin 2, myeloid differentiation factor 88

## Abstract

Autophagy is an important system conserved in eukaryotes that maintains homeostasis by degrading abnormal proteins. Autophagy incompetence in intestinal epithelial cells causes the abnormal function of intestinal stem cells and other cells and damages intestinal barrier function. The disruption of the intestinal barrier causes chronic inflammation throughout the body, followed by impaired glucose and lipid metabolism. *Lactiplantibacillus plantarum* OLL2712 (OLL2712) is a lactic acid bacterium that induces interleukin-10 production from immune cells, alleviates chronic inflammation, and improves glucose and lipid metabolism. In this study, we hypothesized that OLL2712 exerts anti-inflammatory effects by inducing autophagy and ameliorating intestinal barrier dysfunction, and we investigated its autophagy-inducing activities and functions. Caco-2 cells stimulated with OLL2712 for 24 h showed an increased number of autolysosomes per cell, compared with unstimulated cells. Therefore, the permeability of fluorescein isothiocyanate dextran 4000 (FD-4) was suppressed by inducing autophagy. In contrast, mucin secretion in HT-29-MTX-E12 cells was also increased by OLL2712 but not via autophagy induction. Finally, the signaling pathway involved in autophagy induction by OLL2712 was found to be mediated by myeloid differentiation factor 88 (MYD88). In conclusion, our findings suggest that OLL2712 induces autophagy in intestinal epithelial cells via MYD88, and that mucosal barrier function is strengthened by inducing autophagy.

## 1. Introduction

Autophagy is an important system that degrades abnormal intracellular proteins, damaged organelles, and invading microorganisms in the cytoplasm of eukaryotes and is induced by nutrient starvation and cellular stress to maintain intracellular homeostasis [1]. Autophagy deficiency is involved in the onset and progression of various diseases, including neurodegenerative diseases, type 2 diabetes, and cancers [2,3,4]. In intestinal epithelial cells, autophagy deficiency has been reported to disrupt the intestinal barrier function and induce Crohn’s disease [5] by causing abnormalities in intestinal stem cell function, mucin secretion from goblet cells, and antimicrobial peptide secretion from Paneth cells [6,7,8]. Humans contain various commensal gut bacteria. Lactic acid bacteria (LAB) are common commensal bacteria that are well-known as food-fermenting bacteria and have been consumed worldwide since ancient times. Recently, various health-promoting effects of LAB have been reported, such as amelioration of constipation [9], immune modulatory effects [10], and the improvement of mucosal intestinal barrier function [11].

*Lactiplantibacillus plantarum* (basonym: *Lactobacillus plantarum*) OLL2712 is a LAB strain. OLL2712 strongly induces interleukin (IL)-10 production in dendritic cells and macrophages, which, in turn, alleviate chronic inflammation and improve metabolic abnormalities in experimental mice [12,13]. Consuming yogurt containing this strain by healthy but overweight adults inhibits body fat accumulation [14]. In addition, yogurt consumption for 12 weeks by participants with prediabetes improved glucose metabolism and reduced blood HbA1c levels [15]. We attribute these effects to the stimulation of IL-10 production by immune cells because IL-10 is an anti-inflammatory cytokine that inhibits the secretion of inflammatory cytokines and improves glucose uptake in chronically inflamed adipose tissues [16].

On the other hand, previous studies indicated that the disruption of intestinal epithelial barrier function caused intestinal inflammation, induced systemic chronic inflammation, and impaired glucose metabolism by worsening insulin resistance [17]. Another study reported that autophagy ameliorated dextran sodium sulphate-induced colitis via an anti-inflammatory effect [18]. These studies showed that autophagy induction is important for maintaining intestinal homeostasis and suppressing inflammation. LAB have been reported to induce autophagy and improve intestinal barrier function. For example, *Lactobacillus rhamnosus* suppresses barrier disruption caused by *Fusobacterium nucleatum* infection and reduces inflammation by restoring autophagy flux in mice and Caco-2 cells [19]. *Lactobacillus acidophilus*, when administered to live cells rather than heat-treated cells, increased the fecal short-chain fatty acids (SCFAs) concentration in an ulcerative colitis rat model, which resulted in the induction of mitophagy and improved mucosal barrier function [20]. However, these studies did not confirm the autophagy-inducing effect of heat-treated bacteria on intestinal epithelial cells.

OLL2712 suppresses the amount of fluorescein isothiocyanate dextran 4000 (FD-4) permeating into the serum in lean mice and the ileum in high-fat diet-fed mice [21]; however, its mechanism is not clear. The suppressive effect of OLL2712 on chronic inflammation could be because of the production of IL-10 and the induction of autophagy in intestinal epithelial cells. In the present study, we hypothesized that OLL2712 induced autophagy in intestinal epithelial cells and improved intestinal barrier function. We found that OLL2712 induced autophagy and improved intestinal barrier function. We also revealed that the mechanism of autophagy induction was mediated by myeloid differentiation factor 88 (MYD88). Furthermore, other strains of *L. plantarum* and other LAB species also exhibited autophagy-inducing activity. Thus, the ability to induce autophagy is dependent on strains.

## 2. Materials and Methods

### 2.1. Preparation of Bacterial Cells

*Lactiplantibacillus plantarum* OLL2712 was isolated in our laboratory and deposited in the International Patent Organism Depositary (Chiba, Japan) under accession No. FERM BP-11262, which was used in this study. The other bacterial strains are listed in Table 1. They were cultured in de Man–Rogosa–Sharpe broth (MRS; Becton Dickinson, Franklin Lakes, NJ, USA) at 37 °C for 18 h under anaerobic conditions with AnaeroPouch-Anaero (Mitsubishi Gas Chemical, Tokyo, Japan). The bacterial cells were harvested and washed twice with phosphate-buffered saline (PBS; pH 7.2), then washed once with distilled water. The cells were then heat-treated at 75 °C for 60 min and then freeze-dried. The lyophilized cells were resuspended in distilled water at a concentration of 10 mg/mL and used for in vitro assays.

### 2.2. Cell Culture

Caco-2 cells (European Collection of Authenticated Cell Cultures; ECACC, Salisbury, UK) were cultured in high-glucose Dulbecco’s Modified Eagle Medium (DMEM) (Sigma-Aldrich, St. Louis, MO, USA) supplemented with 10% fetal bovine serum (FBS; Biowest, Nuaille, France), 100 U/mL penicillin, 100 µg/mL streptomycin (Gibco, Waltham, MA, USA), and 1% minimum essential medium non-essential amino acid (MEM-NEAA; Sigma-Aldrich) in a 10 cm dish and were maintained at 37 °C in a humidified atmosphere containing 5% CO_2_. They were split at 80% confluence every three or four days. HT-29-MTX-E12 cells (ECACC), goblet cell-like cells, were cultured using the same procedure.

Using permeable supports (Transwell, 12 mm or 6.5 mm diameter, 0.4 mm pore size; Corning, Corning, NY, USA), the Caco-2 and HT-29-MTX-E12 cells were plated at a density of 9 × 10^4^ cells/cm^2^ and cultured for three weeks by changing the medium every two or three days.

### 2.3. Detection of Autophagy Activity Using DALGreen

Caco-2 cells were plated at a density of 1 × 10^4^ cells in a 96-well culture plate and were cultured at 37 °C in 5% CO_2_. DALGreen (Dojindo Laboratories, Kumamoto, Japan) was used to detect autophagy [22], following the manufacturer’s instructions. Briefly, the cells were cultured in the presence of 1 µM DALGreen for 30 min in the case of Caco-2 cells or 3 µM DALGreen for 60 min in the case of Caco-2 monolayers, respectively. The cells were then washed with Hanks’ balanced salt solution (HBSS; Nacalai Tesque, Kyoto, Japan). To stimulate autophagy, heat-treated bacterial cells or other bacterial strains were added at 100 µg/mL. As a positive control, raloxifene (Toronto Research Chemicals, ON, Canada), an inducer of cellular autophagy [23], or bafilomycin A1 (Sigma-Aldrich), an autophagy inhibitor, was added to the cultures at concentrations of 20 µM or 10 nM, respectively. After culturing for 24 h, the nuclei were stained with Hoechst 33342 (Dojindo Laboratories) and examined under a fluorescence microscope (Keyence, BZ-X810, Osaka, Japan). Green fluorescence and Hoechst 33342 staining were detected using a green fluorescent protein (GFP) filter (excitation: 470/40 nm, emission: 525/50 nm) and a 4′,6-diamidino-2-phenylindole (DAPI) filter (excitation: 360/40 nm, emission: 460/50 nm), respectively. Signaling intensities of autolysosomal puncta and cell counts were automatically calculated using a hybrid cell count application in the BZ-X Analyzer software (Keyence).

### 2.4. Developing a Stable GFP-LC3-Expressing Caco-2 Strain

The pCMV-GFP-LC3 vector was purchased from Cell Biolabs (San Diego, CA, USA). The plasmid was isolated using Midi Prep (Qiagen, Hilden, Germany) and linearized using the restriction enzyme *Apa*LI (Takara Bio, Shiga, Japan). Caco-2 cells were cultured up to 70–80% confluence and transfected using Lipofectamine 3000 (Thermo Fisher Scientific, Waltham, MA, USA). The cells were grown in a selective medium containing G-418 at 2.0 mg/mL (InvivoGen, San Diego, CA, USA). After the cells proliferated, they were screened to determine whether the transfection was successful by examining them under a fluorescence microscope, as described in the DALGreen assay, and confirming that GFP-LC3 puncta were stimulated by the positive control, raloxifene. These screenings were repeated three times, and a strain with the highest fluorescence was established.

### 2.5. Detection of Autophagy Activity Using the Stable GFP-LC3-Expressing Caco-2 Strain

The stable GFP-LC3-expressing Caco-2 strain was plated at a density of 1 × 10^4^ cells in a 96-well culture plate. The cells were then stimulated with 20 µM raloxifene or 100 µg/mL OLL2712. After cultivation for 24 h at 37 °C in 5% CO_2_, the nuclei were stained with Hoechst 33342 and observed for the GFP-LC3 puncta, as described in the DALGreen assay.

### 2.6. RNA Isolation and Quantitative Analysis by Real-Time Polymerase Chain Reaction (PCR)

Total RNA was extracted from Caco-2 cells using a Maxwell RSC48 automatic nucleic acid extractor (Promega, Madison, WI, USA) with a Maxwell RSC Simply RNA Cells Kit (Promega), following the manufacturer’s instructions. RNA was quantified and assessed for purity using NanoDrop (Thermo Fisher Scientific). Complementary DNA was synthesized using PrimeScript RT Master Mix (Takara Bio), and PCR was performed using a GeneAmp PCR system 9700 (Applied Biosystems, Waltham, MA, USA). Real-time PCR was performed using the KOD SYBER qPCR Mix (Toyobo, Osaka, Japan) and the QuantStudio 3 Real-time PCR system (Applied Biosystems), according to the manufacturer’s instructions. The primer sets used are listed in Table S1. Amplification conditions were as follows: pre-denaturation at 98 °C for 2 min, denaturation at 98 °C for 10 s, annealing at 60 °C for 10 s, extension at 68 °C for 30 s, a total of 40 cycles, and extension at the melting curve before the end. mRNA expression was normalized to that of a house-keeping gene, *Glyceraldehyde 3-phosphate dehydrogenase* (*GAPDH*).

### 2.7. Western Blotting

To prepare whole-cell extracts, Caco-2 cells were washed with ice-cold PBS, suspended in radioimmunoprecipitation assay buffer (Nacalai Tesque, Kyoto, Japan) containing 10% protease inhibitor cocktail (Sigma-Aldrich), 1% phosphatase inhibitor cocktail (Nacalai Tesque), 1 mM ethylene glycol tetraacetic acid, and 1 mM ethylenediaminetetraacetic acid, then sonicated for 25 s on ice. The protein concentration in the cell lysates was measured using the Pierce BCA Protein Assay Kit (Thermo Fisher Scientific). They were mixed with a 2× or 4× sample buffer (1× concentration: 62.5 mM Tris-HCl (pH 6.8), 5% (*v*/*v*) β-mercaptoethanol, 2% (*w*/*v*) sodium dodecyl sulphate (SDS), 5% (*w*/*v*) sucrose, and 0.005% (*w*/*v*) bromophenol blue) and boiled for 5 min. Equivalent amounts of the protein were loaded onto SDS-polyacrylamide gels and transferred to a 0.2 µm polyvinylidene difluoride membrane. The membranes were blocked with 5% bovine serum albumin (Fujifilm Wako Pure Chemical) in Tris-buffered saline (TBS-T; 50 mM Tris-HCl (pH 7.4), 138 mM NaCl, 2.7 mM KCl, and 0.05% Tween-20). The membranes were washed three times with TBS-T, then incubated at 4 °C overnight or room temperature for 1 h, with a primary antibody, anti-light chain 3B (LC3B) (ab48394, 1:500, Abcam, Cambridge, UK) or horseradish peroxidase (HRP) anti-beta actin (ab20272, 1:5000, Abcam). The LC3B membrane was subsequently washed three times with TBS-T and incubated for 1 h with HRP-conjugated anti-rabbit IgG secondary antibodies (7074S, 1:1000, Cell Signaling Technology, Danvers, MA, USA). Proteins were detected by enhanced chemiluminescence using Amersham ECL (GE Healthcare Bioscience, Westborough, MA, USA). The intensities of the bands were quantified using the ImageJ Fiji software v1.53f51 [24]. Autophagy induction was measured by calculating the LC3-II/β-actin ratio.

### 2.8. Simple Western Analysis

WES, an automated capillary-based electrophoresis system (ProteinSimple, San Jose, CA, USA) was used to analyze protein levels, following the manufacturer’s instructions. Briefly, cell lysates were prepared using the above-mentioned methods and diluted with the 0.1× sample buffer to a protein concentration of 1 mg/mL. Then, the lysates were mixed with the 5× fluorescent master mix in a 4:1 ratio, and the mixtures were heated at 95 °C for 5 min. The prepared lysates, antibody dilution buffer, primary antibody, anti-mucin 2 (MUC2) antibody (ab134119, 1:50, Abcam), HRP-conjugated secondary rabbit antibody, and chemiluminescent substrate mix (luminol:peroxide mixture in a 1:1 ratio) were dispensed into predetermined wells in an assay plate. For the total protein assay, the total protein label reagent and total protein streptavidin-HRP were used instead of the primary antibody and the HRP-conjugated secondary antibody. After centrifugation, a wash buffer was added to the plate. The plate and capillary cartridge were placed into a WES instrument (ProteinSimple), and the runs were started. After the analysis, the resulting data were evaluated using the Compass software v3.1.7 (ProteinSimple). MUC2 levels were normalized to that of the total protein content.

### 2.9. FD-4 Permeability Test

Caco-2 monolayers were used for FD-4 permeability tests. The cells were stimulated with 20 µM raloxifene, 100 µg/mL OLL2712, or 100 µg/mL OLL2712 in the presence of 10 nM bafilomycin A1. After cultivation for 24 h, the cells were washed and incubated with HBSS at 37 °C in 5% CO_2_ for 30 min. The cells were then incubated with HBSS containing 1 mg/mL FD-4 solution (Sigma-Aldrich) for 1 h. The FD-4 permeability was evaluated using 100 μL of the culture medium from the basolateral side. A Synergy H1 microplate reader (BioTek Instruments, Winooski, VT, USA) with a 485 nm excitation and 535 nm emission filters was used to measure the fluorescence signal. Standard curves were prepared using a dilution series of FD-4 solutions to determine the concentration of the permeated FD-4.

### 2.10. MUC2 Enzyme-Linked Immunosorbent Assay (ELISA)

HT-29-MTX-E12 monolayers were cultured in the same manner as Caco-2 monolayers. The cells were incubated with 100 µg/mL OLL2712 in the absence or presence of 10 nM bafilomycin A1 for 72 h. Then, the cells and culture supernatants were collected by pipetting, and the centrifuged supernatants were used for the assay. The MUC2 concentration in the supernatant was determined using a Human MUC2 ELISA Kit (MyBioSource, San Diego, CA, USA), according to the manufacturer’s instructions. The quantified mucin concentration was normalized to the protein concentration in the supernatant.

### 2.11. Small Interfering RNA (siRNA) Knockdown Experiments

MYD88 siRNA was purchased from Thermo Fisher Scientific and transfected into Caco-2 cells using the Lipofectamine RNAiMAX transfection reagent (Thermo Fisher Scientific), according to the manufacturer’s instructions. Briefly, Caco-2 cells were plated in a 96-well plate, with antibiotic-free DMEM supplemented with 10% FBS and 1% MEM-NEAAs, and cultured overnight. After the cells reached approximately 50% confluence, the siRNA and Lipofectamine RNAiMAX complexes were added to the culture plates. After 48 h of transfection, the cells were washed and used for the assay.

### 2.12. Statistical Analysis

Data are presented as the mean value ± standard error. When the data followed equal variances, the difference between the two groups was analyzed using Student’s *t*-test. Otherwise, Welch’s *t*-test was used. Statistical significance was set up at *p* < 0.05.

## 3. Results

### 3.1. OLL2712 Increases Autophagy in Intestinal Epithelial Cells

**Figure 1:**
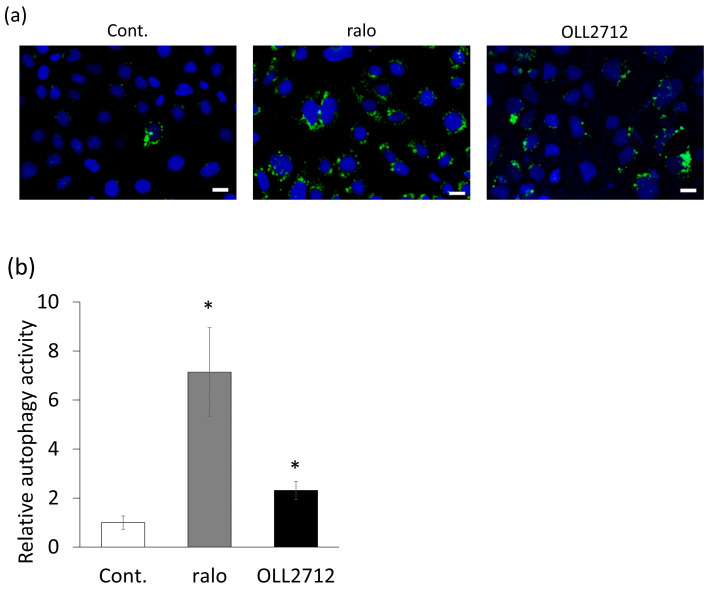
*Lactiplantibacillus plantarum* OLL2712 (OLL2712) increased autolysosome puncta in intestinal epithelial cells. (**a**) Fluorescent microscopic images of autolysosome puncta in Caco-2 cells detected with DALGreen and (**b**) the fluorescent intensities of autolysosomes per cell (n = 10). Scale bars were 20 µm. Data are shown as the relative level to the control. *: *p* < 0.05 vs. control. Cont.: control; ralo: raloxifene.

We first examined whether OLL2712 induced autophagy in the steady state of intestinal epithelial cells using DALGreen. Caco-2 cells were stimulated with 20 µM raloxifene or 100 µg/mL OLL2712 for 24 h, after pre-incubation with DALGreen for 30 min. They were examined under a fluorescence microscope. DALGreen detects autolysosomes, which are generated by the fusion of autophagosomes with lysosomes, as green fluorescent puncta. The fluorescence of the positive control, raloxifene, was seven-fold higher than that of the control, confirming that raloxifene exerted a positive effect. We found that OLL2712 increased the number of autolysosomes per cell by approximately two-fold in Caco-2 cells, compared with that in the control, after 24 h (Figure 1). We further examined whether the activity could be observed using Caco-2 monolayers, such as small intestinal epithelial cells, and found increased activity to the same extent as that for raloxifene (Figure S1).

**Figure 2:**
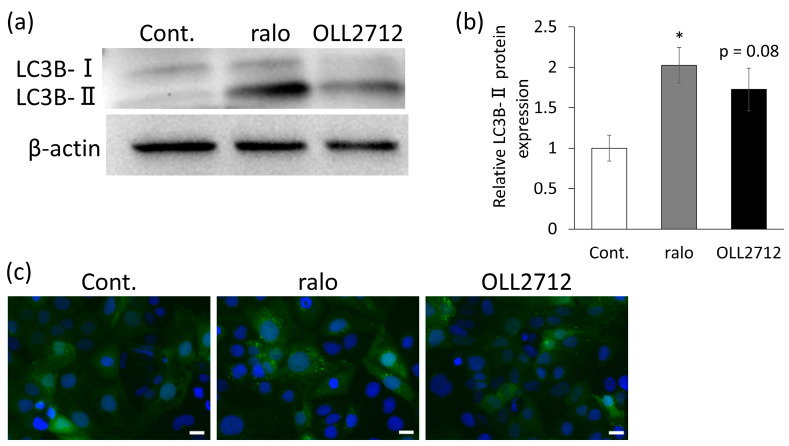
OLL2712-induced light chain 3 (LC3) expression. (**a**) Western blots of LC3B-I and II and (**b**) the relative LC3B-II/β-actin ratio to the control (n = 4). β-actin was used as a loading control. (**c**) Fluorescent microscopic images of green fluorescent protein (GFP)-LC3 puncta in stable GFP-LC3-expressing Caco-2 cells. GFP-LC3 puncta and nuclei are shown in green and blue, respectively. Scale bars were 20 µm. *: *p* < 0.05 vs. control. Cont.: control; ralo: raloxifene.

LC3B is a well-known marker of autophagy. LC3B-I is the cytosolic form of LC3, which turns into LC3B-II when it is conjugated to phosphatidylethanolamine. LC3B-II is recruited to the autophagosomal membrane and is, therefore, an important indicator of autophagic activity [25,26]. To determine LC3B protein levels, we performed Western blotting. Caco-2 cells were stimulated with 20 µM raloxifene or 100 µg/mL OLL2712 for 24 h. The level of LC3B-II significantly increased, owing to raloxifene stimulation, and tended to increase, owing to OLL2712 stimulation (*p* = 0.08; Figure 2a,b). Furthermore, we used genetically modified Caco-2 cells that constitutively expressed GFP-LC3 to detect the number of LC3 puncta more accurately and concisely. In this assay, the activity could be detected by the fluorescence emitted when GFP-conjugated LC3 was present on the isolation membrane of the autophagosome. The results showed that, compared with the control, cells stimulated with OLL2712 showed GFP-LC3 puncta (Figure 2c), indicating the autophagy-inducing activity of OLL2712.

**Figure 3:**
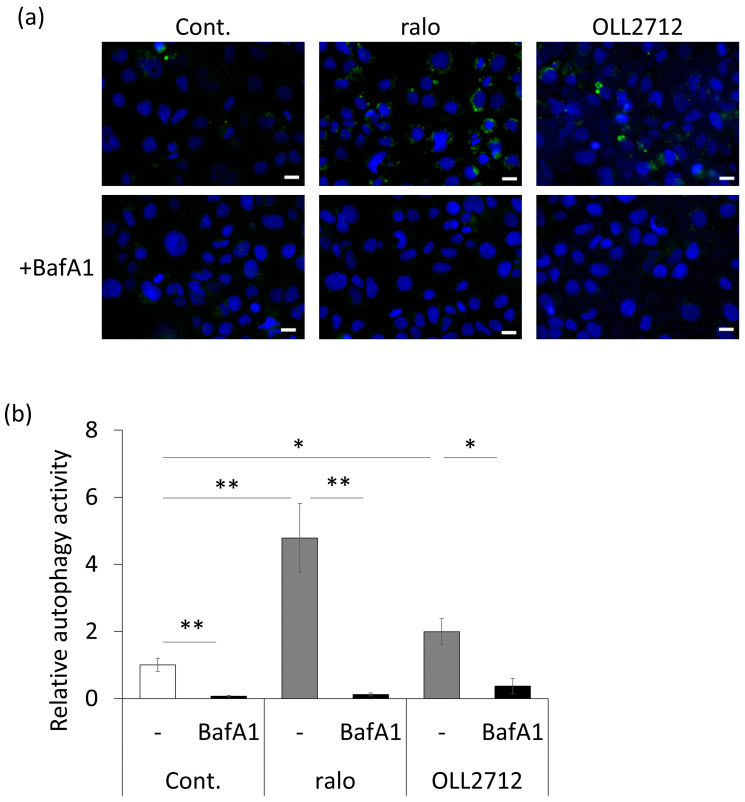
Autophagy induced by OLL2712 was suppressed by the autophagy inhibitor bafilomycin A1. (**a**) Fluorescent microscopic images of autolysosome puncta in Caco-2 cells, in which autophagy was inhibited by bafilomycin A1 and was detected with DALGreen, and (**b**) the fluorescent intensities of autolysosomes per cell (n = 7–10). Scale bars were 20 µm. Data are shown as the relative level to the control. *: *p* < 0.05, **: *p* < 0.01. Cont.: control, ralo: raloxifene, BafA1: bafilomycin A1.

Bafilomycin A1 is an autophagy inhibitor that inhibits the fusion of autophagosomes and lysosomes [27]. We examined whether inducing autophagy using 100 µg/mL OLL2712 was suppressed by 10 nM bafilomycin A1. This substantially suppressed the number of autolysosomes induced by OLL2712 (Figure 3). Here, we observed that cell death was not induced by stimulation with OLL2712. It was confirmed that inducing autophagy using OLL2712 was due to neither apoptosis nor necrosis induction to the cells (Figure S2). These results also indicated that OLL2712 induced autophagy in intestinal epithelial cells.

### 3.2. Autophagy Induced by OLL2712 in Intestinal Epithelial Cells Strengthens the Mucosal Barrier

**Figure 4:**
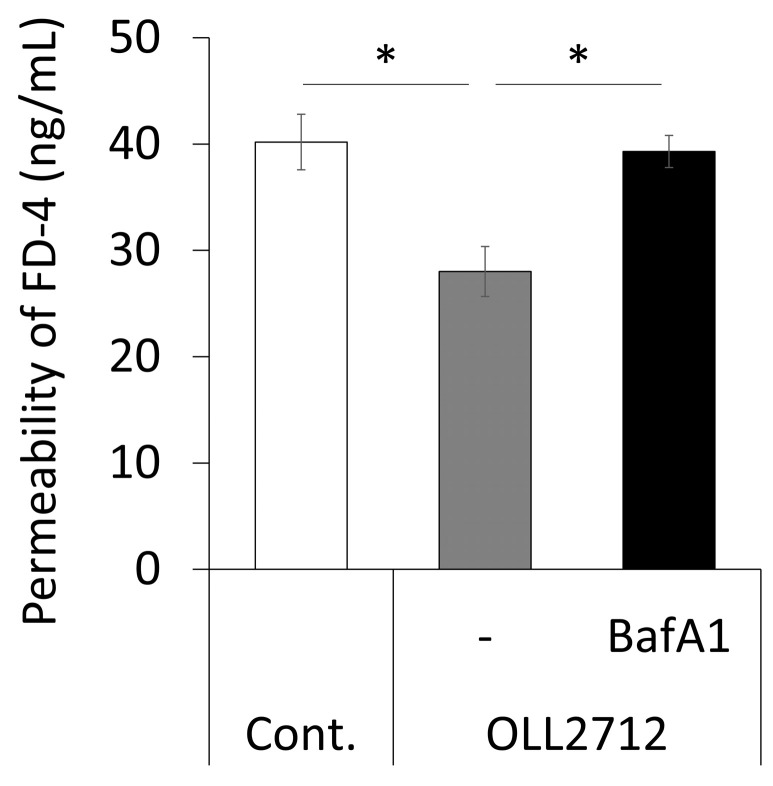
OLL2712 promoted intestinal barrier function via autophagy. Permeated fluorescein isothiocyanate dextran 4000 levels (FD-4) across Caco-2 monolayers stimulated with OLL2712 in the absence or presence of bafilomycin A1 for 24 h. The fluorescent intensity of the basolateral side was measured (n = 3, 4). *: *p* < 0.05 vs. OLL2712. Cont.: control, BafA1: bafilomycin A1.

Some LAB can improve intestinal barrier function both in vitro and in vivo [11,28,29]. The relationship between autophagy and intestinal barrier function showed that the activation of autophagy strengthened tight junctions [30]. Therefore, we investigated whether the induction of autophagy by OLL2712 affected intestinal barrier function. We tested the FD-4 permeability from the apical side to the basolateral side using Caco-2 monolayers. They were stimulated with OLL2712 in the absence or presence of bafilomycin A1 for 24 h; then, they were challenged with FD-4. The result revealed that OLL2712 suppressed FD-4 permeation, whereas bafilomycin A1 inhibited this effect (Figure 4). In an independent experiment, the addition of bafilomycin A1 alone had no effect on the permeation (Figure S3a). This suggests that autophagy activation by OLL2712 contributes to the strengthening of the mucosal barrier in Caco-2 cells.

**Figure 5:**
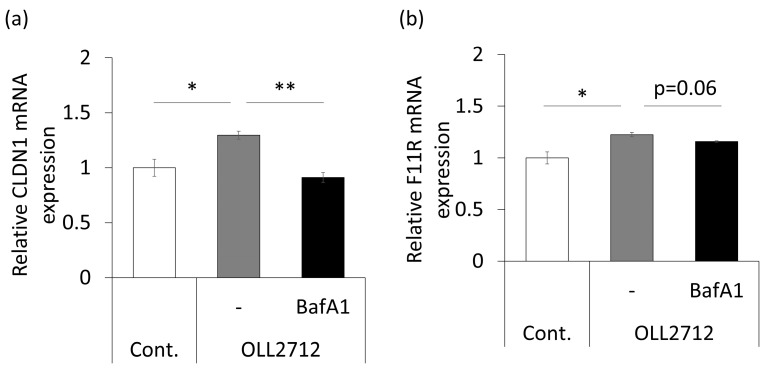
OLL2712 increased tight junction-related gene expression via autophagy. (**a**,**b**) Relative mRNA expression of the tight junction-related gene in Caco-2 monolayers. Total mRNA was extracted from the Caco-2 monolayers after 9 h of stimulation. (**a**) *CLDN1* and (**b**) *F11R* were normalized with *GAPDH,* and data are shown as the relative expression to the control (n = 3). *: *p* < 0.05, **: *p* < 0.01 vs. OLL2712. Cont.: control, BafA1: bafilomycin A1.

Next, we analyzed expressions of tight junction-related genes. Caco-2 monolayers were stimulated with the same conditions described above for 9 h. The results showed that claudin-1 (*CLDN1*) and the junctional adhesion molecule-A (JAM-A/*F11R*) were significantly upregulated by the strain, whereas the effect was significantly suppressed or tended (*p* = 0.06) to be suppressed by the addition of bafilomycin A1 (Figure 5). The expression of other tight junction-related genes, such as occludin (*OCLN*), zonula occludens-1 (ZO-1/*TJP1*), and *CLDN4* did not change significantly (Figure S3b–d). These results showed that OLL2712 strengthened intestinal barrier function by stimulating tight junction-related mRNA expression via autophagy.

### 3.3. OLL2712 Promotes Mucin Secretion in an Autophagy-Independent Pathway

**Figure 6:**
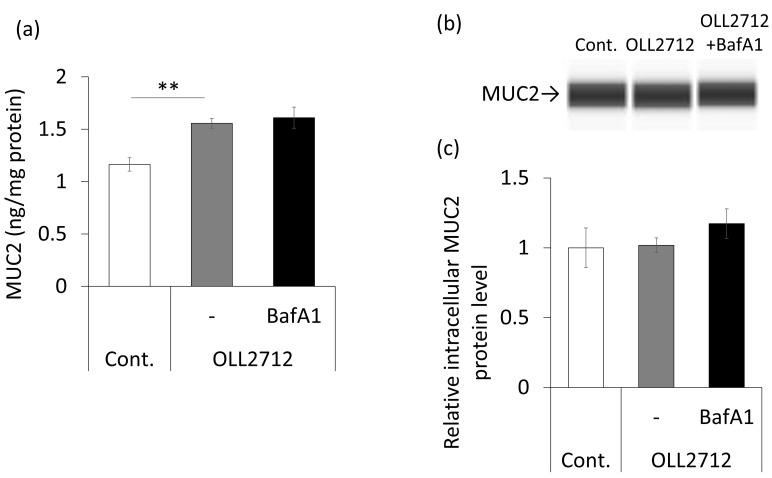
OLL2712 promoted mucin secretion independent of autophagy. (**a**) Mucin 2 (MUC2) concentration in the culture supernatant of the HT-29-MTX-E12 cell line. The MUC2 concentration was normalized with the supernatant protein concentration (n = 4). (**b**) Simple Western images for the intracellular MUC2 levels and (**c**) the level of MUC2 normalized with that of the total protein. Data are shown as the relative level to the control (n = 4). **: *p* < 0.01 vs OLL2712. Cont.: control, BafA1: bafilomycin A1.

The mucus layer on intestinal epithelial cells constitutes a physical barrier in vivo [31]. To investigate whether OLL2712 affects mucin secretion in goblet cells via autophagy, we quantified mucin levels by ELISA after 72 h of stimulation in HT-29-MTX-E12 cells, a cell line that secretes mucin-like goblet cells [32]. Mucin secretion was significantly increased by OLL2712 stimulation; however, the autophagy inhibitor did not suppress mucin secretion (Figure 6a). OLL2712 promoted mucin secretion independent of autophagy. Additionally, although intracellular MUC2 levels were examined by Western blotting, there were no significant differences between the groups (Figure 6b,c). Therefore, we assumed that OLL2712 promoted extracellular mucin secretion, rather than enhancing its differentiation into goblet cells.

### 3.4. OLL2712 Induces Autophagy via MYD88

**Figure 7:**
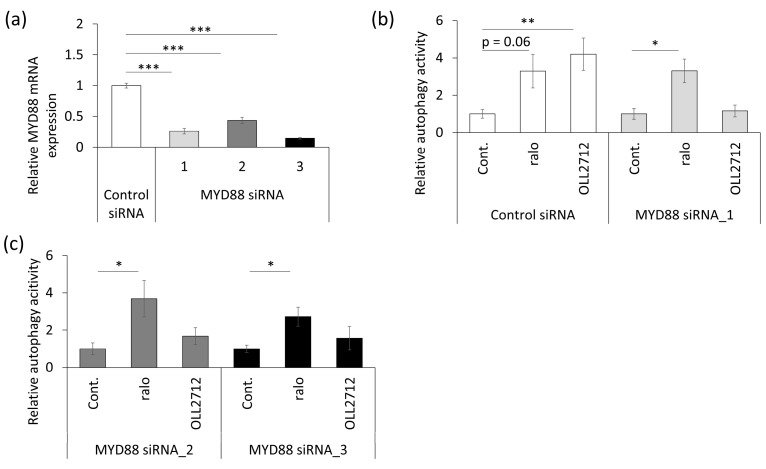
MYD88 mediated OLL2712-induced autophagy. (**a**) Relative expression of *MYD88* in Caco-2 cells transfected with three types of small interfering RNAs specific for *MYD88* (MYD88 siRNA, n = 3). *MYD88* were normalized with *GAPDH,* and data are shown as the relative expression to the control siRNA. (**b**,**c**) Fluorescent intensity of autolysosomes per cell detected with DALGreen. Data are shown as the relative level to the control (n = 4–10). *: *p* < 0.05, **: *p* < 0.01, ***: *p* < 0.001. Cont.: control, ralo: raloxifene.

MYD88 is an adapter factor downstream of toll-like receptors (TLRs) and is involved in various signaling pathways, such as immune and inflammatory responses [33]. A previous study reported that MYD88 was required for autophagy induction in mouse small intestinal epithelial cells [34]. We hypothesized that autophagy activation by OLL2712 was also mediated by MYD88. To assess the involvement of MYD88, transient MYD88 knockdown was performed in Caco-2 cells using siRNA. First, we confirmed the effects of three types of MYD88 siRNAs used to knock down different sequences. The results showed that MYD88 expression was suppressed by 60% or more (Figure 7a). Second, we confirmed whether MYD88 knockdown inhibited autophagy promotion by OLL2712. Transient MYD88 knockout cells or control siRNA-transfected cells were stimulated with raloxifene or OLL2712 for 24 h. Control siRNA did not attenuate OLL2712-induced autophagy, whereas all three siRNAs inhibited it (Figure 7b,c). We verified that the siRNA transfection did not induce cytotoxicity (Figure S4). These results indicated that there were no off-target effects, and that OLL2712 activated autophagy via MYD88.

### 3.5. Some LAB Strains Promote Autophagy in Caco-2 Cells

**Figure 8:**
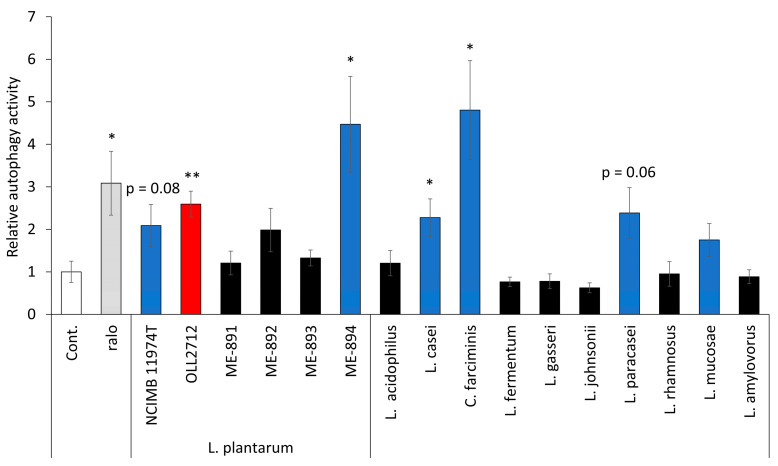
Some lactic acid bacteria (LAB) promoted autophagy in Caco-2 cells. Relative autophagy activity induced by LAB. The fluorescence intensity of autolysosomes per cell was determined from the microscopic images. The red bar indicates OLL2712. Blue bars indicate LAB capable of activating autophagy (n = 8–10). Data are shown as the relative level to the control. *: *p* < 0.05, **: *p* < 0.01 vs control. Cont.: control, ralo: raloxifene.

To investigate whether the effect was specific to OLL2712, other strains belonging to *L. plantarum* or other LAB species were also evaluated for their autophagy activation abilities. Caco-2 cells were cultured with LAB species, as listed in Table 1, for 24 h after pre-incubation with DALGreen. Then, they were examined under a fluorescence microscope. In *L. plantarum,* apart from OLL2712, other strains, including the type strain, induced autophagy (Figure 8). OLL2712 showed slightly higher activity than the type strain of *L. plantarum*, NCIMB 11974^T^. Regarding other species of LAB, type strains of *Lacticaseibacillus casei, Companilactobacillus farciminis, Lacticaseibacillus paracasei,* and *Limosilactobacillus mucosae* also promoted autophagy. *C. farciminis* ATCC 29644^T^ and *L. plantaum* ME-894 showed higher activity than OLL2712. The activities of the other strains were less than or equal to those of OLL2712. We found that there were some LAB that induced autophagy other than OLL2172 in Caco-2 cells, and the activity was dependent on strains.

## 4. Discussion

Previous studies have investigated the ability of LAB to induce autophagy in intestinal epithelial cells and its effects on intestinal barrier function. Duan et al. reported that *L. rhamnosus* induced autophagy and inhibited barrier disruption in the *Fusobacterium nucleatum* infection mouse model and Caco-2 cells. However, they did not provide direct evidence that the induced autophagy was a causal factor in the inhibition of barrier disruption [19]. In another study, Li et al. demonstrated that it was not heat-treated bacterial cells but live *L. acidophilus* that increased the concentration of fecal SCFAs, which protected intestinal barrier function via mitophagy [20]. The induction of autophagy by bacterial cells was not observed. In this study, we demonstrated that OLL2712 stimulated autophagy in Caco-2 cells and promoted intestinal barrier function. Moreover, it was also revealed that MYD88 was involved in the mechanism of autophagy induction by OLL2712.

OLL2712 activated autophagy in intestinal epithelial cells, as evidenced by an increase in autolysosomes and the autophagy marker LC3B-II. We also confirmed that OLL2712 did not induce apoptosis or necrosis accompanying autophagy (Figure S2). In addition, although autophagy has been reported to be related to mitosis [35], whether mitosis regulates autophagy or not or if it is the opposite is still under debate. Further experiments will be required to clarify the mechanism in further detail. On the other hand, the induction of autophagy effects by OLL2712 suppressed FD-4 permeability. An improvement in intestinal barrier function increased glucose and lipid metabolism and suppressed inflammation [17]. Therefore, the induction of autophagy by OLL2712 could be attributed to the mechanisms involved in its anti-inflammatory effects, followed by improvements in both glucose and lipid metabolism, as observed in both animal experiments and clinical trials [13,14,15]. Nighot et al. reported that the induction of autophagy in intestinal epithelial cells increased trans-epithelial electrical resistance (TEER) and inhibited substance permeability [30]. Interestingly, the stimulation of Caco-2 cells by OLL2712 did not result in any changes in TEER in our study (Figure S3e). TEER is an indicator of ion transport through intracellular gaps; however, it is only relevant under leaky conditions [36]. Under conditions where the barrier function is not damaged, TEER may not be used as a barrier strength indicator. Thus, it is conceivable that FD-4 permeability is more likely to reflect intestinal barrier function [37]. It has already been reported that rapamycin, an autophagy inducer similar to raloxifene, protects against tumor necrosis factor α-induced barrier disruption in Caco-2 cells by inhibiting the mechanistic target of rapamycin (mTOR) signaling [38,39]. In contrast, our study showed that raloxifene did not suppress FD-4 permeability in the steady state. Regarding the possible explanation for the result, there was another report that elucidated the mechanisms of the activation of autophagy, independent of mTOR signaling [40]. Some substances, including raloxifene, stimulate sirtuin1 and/or adenosine monophosphate-activated protein kinase (AMPK)-related pathways [41,42]. Therefore, we speculate that sirtuin1 and/or AMPK pathways are the main pathways that induce autophagy after raloxifene stimulation, and these pathways do not strengthen the barrier function in the steady state.

Mucin is secreted by goblet cells and plays a crucial role as a physical barrier that segregates the intestinal epithelium and commensal bacteria [43]. HT-29-MTX-E12 cells used in this study are goblet-like cells with the ability to secrete mucin [32]. The mucin concentration in the culture supernatant was increased because of OLL2712 stimulation; however, it was not suppressed by the autophagy inhibitor bafilomycin A1. Therefore, the promotion of mucin secretion by OLL2712 stimulation was not related to the activation of autophagy. A previous study reported that heterozygous Atg5, an autophagy-related gene, made the mucin layer thin [7], whereas another study reported that hypomorphic Atg16l1, an important gene for autophagosome formation, had no effect on mucin levels [8]. Therefore, the relationship between autophagy and mucin secretion remains to be elucidated. Regarding the stimulation of mucin production by LAB, lipoteichoic acid (LTA) from *L. paracasei* increases the secretion via TLR2 [44]. Both *L. plantarum* and *L. paracasei* are gram-positive bacteria, which contain LTA as a component of their cell walls [45]. *L. plantarum* should have also promoted mucin secretion via TLR2 in Caco-2 cells, without mediation by autophagy.

MYD88 is an adaptor protein downstream of TLR2 and TLR4 [46]. We found that the MYD88 signaling pathway was important for inducing autophagy in Caco-2 cells by OLL2712. Although both TLR2 and TLR4 are involved in autophagy induction in immune cells, such as macrophages [47,48], no reports on their roles in Caco-2 cells are available. Our results suggest that these receptors also function in Caco-2 cells because they have been reported to be expressed in Caco-2 cells [49]. We expected that OLL2712 or LTA from OLL2712 induced autophagy by TLR2-MYD88 signalings. However, inducing mucin secretion stimulated by OLL2712, which is supposed to be caused via TLR2, was found to be independent of autophagy. To clarify whether the difference between these physiological effects can be caused by the same signaling pathway, further experiments are needed. Regarding the MYD88 signaling pathway, MYD88 activates mixed-lineage kinase 3, which phosphorylates AMPK [50,51]. It has also been reported that AMPK activation induces autophagy, as mentioned earlier [41,42,52]. Therefore, it is assumed that OLL2712 activates AMPK via MYD88 and induces autophagy. Furthermore, AMPK is involved in improving mucosal barrier function via TLR2 or caudal-type homeobox 2 mediations [53,54]. For the reasons presented above, we hypothesized that OLL2712 activated AMPK and autophagy via MYD88 in Caco-2 cells. Further experiments are required to elucidate the precise signaling pathways involved in this activity.

Finally, we observed that some LAB other than OLL2712 could also induce autophagy in Caco-2 cells at different intensities from species to species and strain to strain. Previous reports have shown that the induction of cytokine production by immune cells differs between strains. For example, Toshimitsu et al. showed the difference in the stimulation of IL-10 production using both mice peritoneal macrophages and bone-marrow dendritic cells [13], whereas Kobayashi et al. observed it in IL-23 production from cell-lined mice dendritic cells [55]. Because this study was performed using heat-treated bacteria, we presumed that the reasons for the differences in activity among the strains were the differences in the structure and composition of the cell wall components, including LTA.

In conclusion, we demonstrated that OLL2712 induced autophagy via MYD88 in Caco-2 cells and contributed to the strengthening of mucosal barrier function. These results suggest that its autophagy-inducing ability is associated with mechanisms that impart anti-inflammatory effects, followed by improvement, in both the glucose and lipid metabolisms of this beneficial strain.

## Figures and Tables

**Table 1:** List of lactic acid bacterial strains used in this study.

Species	Strains
*Lactiplantibacillus plantarum* subsp. *Plantarum* *	NCIMB 11974^T^
*Lactiplantibacillus plantarum* *	OLL2712
*Lactiplantibacillus plantarum* *	ME-891 **
*Lactiplantibacillus plantarum* *	ME-892 **
*Lactiplantibacillus plantarum* *	ME-893 **
*Lactiplantibacillus plantarum* *	ME-894 **
*Lactobacillus acidophilus*	JCM 1132^T^
*Lacticaseibacillus casei* *	JCM 1134^T^
*Companilactobacillus farciminis* *	ATCC 29644^T^
*Limosilactobacillus fermentum* *	NRIC 1752^T^
*Lactobacillus gasseri*	JCM 1131^T^
*Lactobacillus johnsonii*	JCM 2012^T^
*Lacticaseibacillus paracasei* *	NBRC 15889^T^
*Lacticaseibacillus rhamnosus* *	JCM 1136^T^
*Limosilactobacillus mucosae* *	NCIMB 13705^T^
*Lactobacillus amylovorus*	JCM 1126^T^

* All former genera are *Lactobacillus.* ** These bacterial strains were obtained from Meiji Co., Ltd. (Tokyo, Japan).

## Data Availability

The data presented in this study are available upon request to the corresponding author.

## Supplementary Materials

The following supporting information can be downloaded at: https://www.mdpi.com/article/10.3390/nu15122655/s1, Figure S1: OLL2712 increased autophagy in Caco-2 monolayers. Figure S2: OLL2712 did not induce cell death of Caco-2 cells. Figure S3: The effects of OLL2712 and other reagents related to autophagy on intestinal barrier function. Figure S4: Small interfering RNA (siRNA) had no effect on cell viability; Table S1: Primer sequences in real-time polymerase chain reaction (PCR) used in this study; Supplementary experimental procedure. References [56,57,58] are cited in the supplementary materials.
