# Supplementary material for: Lactiplantibacillus plantarum OLL2712 Induces Autophagy via MYD88 and Strengthens Tight Junction Integrity to Promote the Barrier Function in Intestinal Epithelial Cells
Source: Nutrients. 2023 Jun 7;15(12):2655. doi: 10.3390/nu15122655 (PMC10300898; doi:10.3390/nu15122655)
Supplement: Supplementary file 1 [file nutrients-15-02655-s001.zip › Supplementary_data.pdf]

Supplementary data

Supplementary experimental procedure

Cell death rate

Caco-2 cells were plated at a density of 1 × 10^4 cells in a 96-well culture plate at 37°C in 5% CO_2. Heat-treated OLL2712 were added at 100 µg/mL and cultured for 24 h. The cells were washed twice with Hanks' balanced salt solution, and stained with propidium iodide (PI) and Hoechst33342 for 30 min at 37°C. The cells were examined under a fluorescence microscope (Keyence, BZ-X810). PI and Hoechst 33342 were detected using TRITC (excitation: 545/25 nm, emission: 605/70 nm, Keyence) and DAPI filters (excitation: 360/40 nm, emission: 460/50 nm, Keyence), respectively. The cell death rate was calculated as the percentage of PI-positive cells among all cells.

Lactate dehydrogenase (LDH) assay

Cell viability was assessed by measuring the release of LDH using Cytotoxicity LDH Assay Kit-WST (Dojindo Laboratories) according to the manufacturer's instructions.

Table S1. Primer sequences in real-time polymerase chain reaction (PCR) used in this study

Gene		Sequences (5′→3′)
GAPDH	Takara, HA067812	
CLDN1 [56]	Forward	GTGCGATATTTCTTCTTGCAGG
	Reverse	TTCGTACCTGGCATTGACTGG
F11R	Forward	ACCTTCTTGCCAACTGGTATCA
	Reverse	AGCACGATGAGCTTGACCTTG
MYD88	Forward	GGCTGCTCTCAACATGCGA
	Reverse	CTGTGTCCGCACGTTCAAGA
OCLN [57]	Forward	TCAGGGAATATCCACCTATCACTTCAG
	Reverse	CATCAGCAGCAGCCATGTACTCTTCAC
TJP1	Forward	ACCAGTAAGTCGTCCTGATCC
	Reverse	TCGGCCAAATCTTCTCACTCC
CLDN4 [56]	Forward	GGCTGCTTTGCTGCAACTGTC
	Reverse	GAGCCGTGGCACCTTACACG

Not referenced PCR primer sequences were obtained from Primerbank (https://pga.mgh.harvard.edu/primerbank/) [58].

Supplementary References

56. Holczbauer, Á.; Gyöngyösi, B.; Lotz, G.; Szijártó, A.; Kupcsulik, P.; Schaff, Z.; Kiss, A. Distinct claudin expression profiles of hepatocellular carcinoma and metastatic colorectal and pancreatic carcinomas. J. Histochem. Cytochem. 2013, 61, 294–305. https://doi.org/10.1369/0022155413479123.
57. Braniste, V.; Leveque, M.; Buisson-Brenac, C.; Bueno, L.; Fioramonti, J.; Houdeau, E. Oestradiol decreases colonic permeability through oestrogen receptor beta-mediated up-regulation of occludin and junctional adhesion molecule-A in epithelial cells. J. Physiol. 2009, 587, 3317–3328. https://doi.org/10.1113/jphysiol.2009.169300.
58. Spandidos, A.; Wang, X.; Wang, H.; Seed, B. PrimerBank: a resource of human

and mouse PCR primer pairs for gene expression detection and quantification. Nucleic Acids Res. 2010, 38, D792–799. https://doi.org/10.1093/nar/gkp1005.
