# Supplementary figures and images for: Lactiplantibacillus plantarum OLL2712 Induces Autophagy via MYD88 and Strengthens Tight Junction Integrity to Promote the Barrier Function in Intestinal Epithelial Cells
Source: Nutrients. 2023 Jun 7;15(12):2655. doi: 10.3390/nu15122655 (PMC10300898; doi:10.3390/nu15122655)

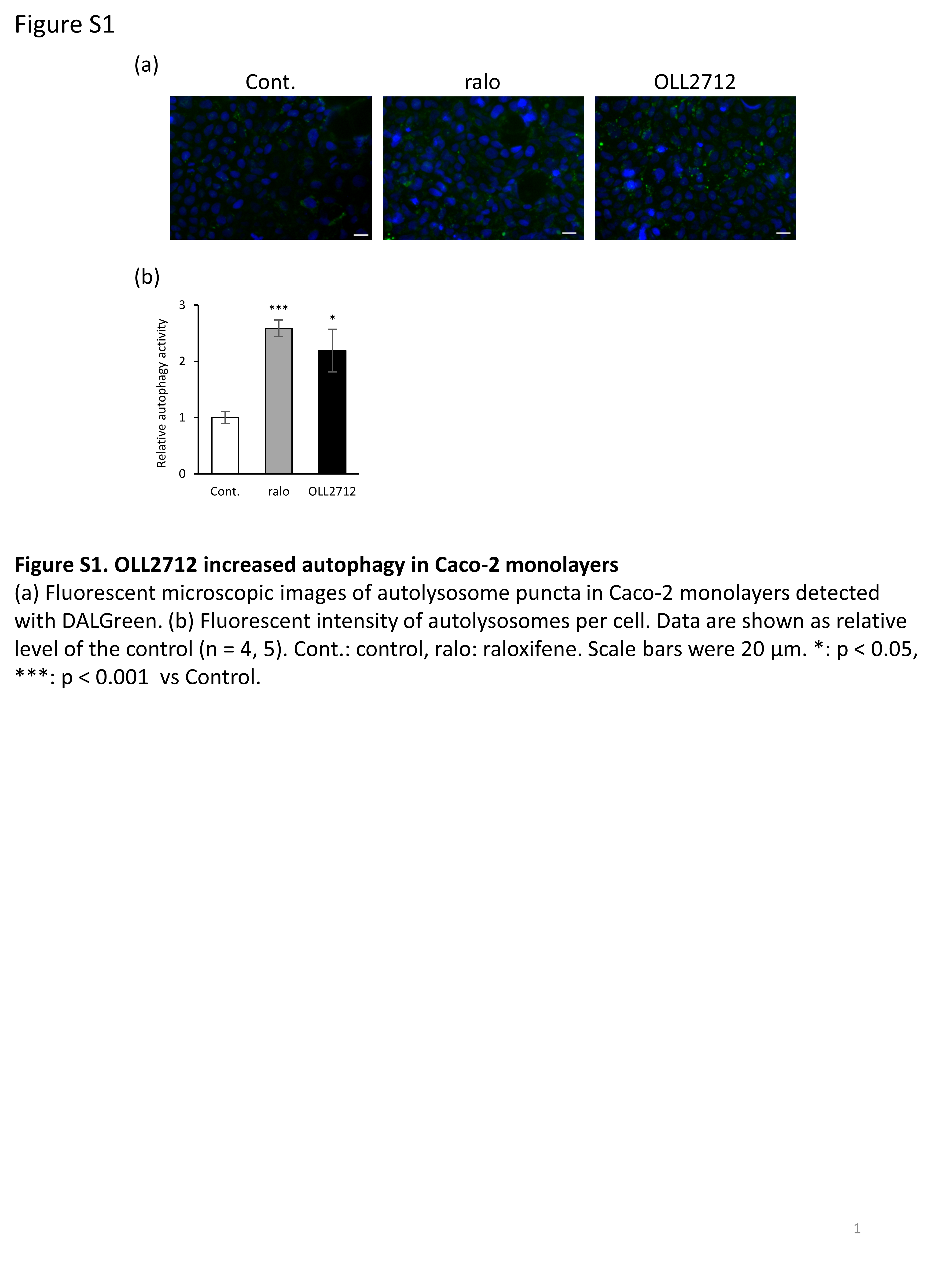

Supplement: Supplementary file 1 [file nutrients-15-02655-s001.zip › Figure S1.TIF]

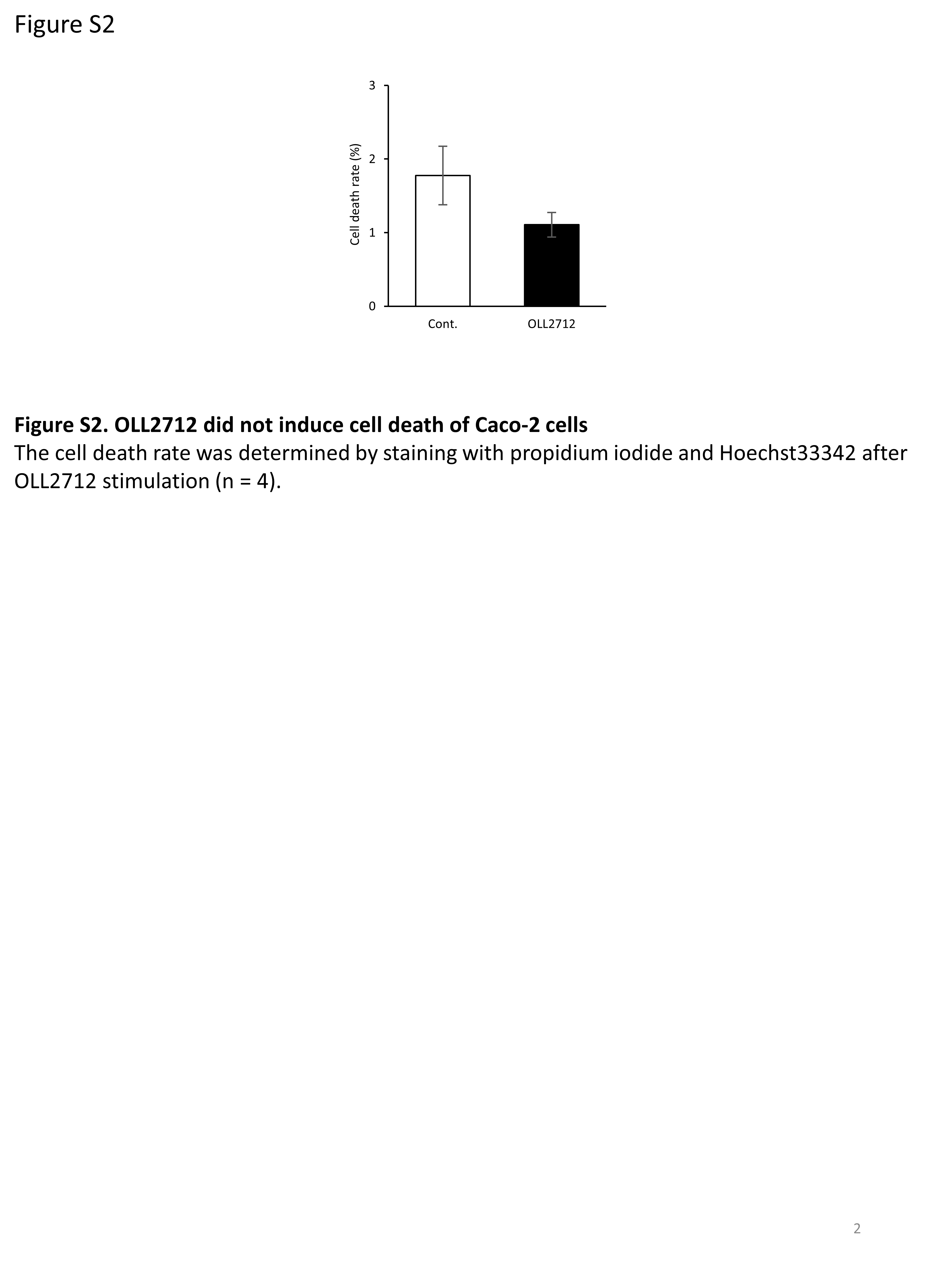

Supplement: Supplementary file 1 [file nutrients-15-02655-s001.zip › Figure S2.TIF]

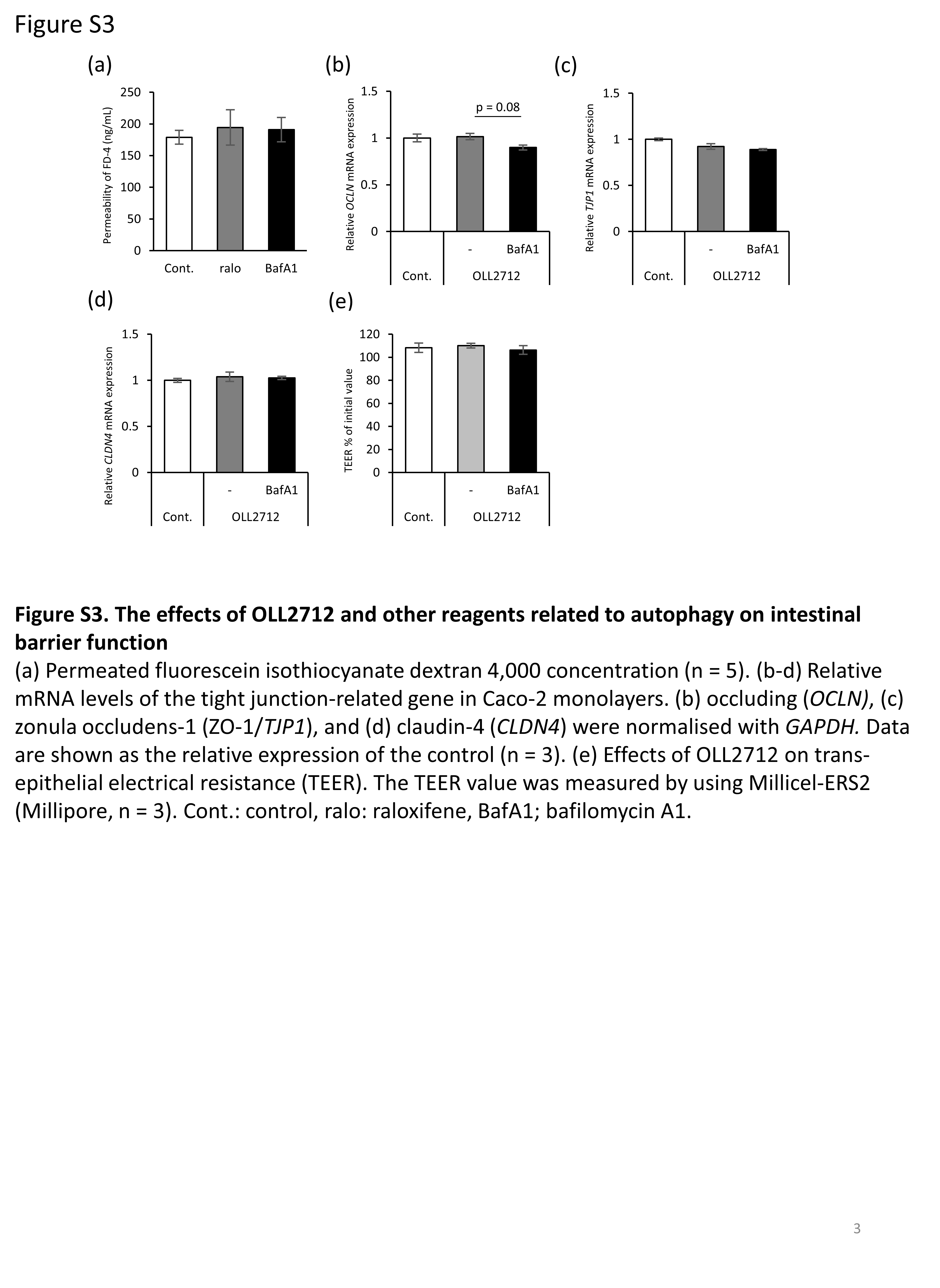

Supplement: Supplementary file 1 [file nutrients-15-02655-s001.zip › Figure S3.TIF]

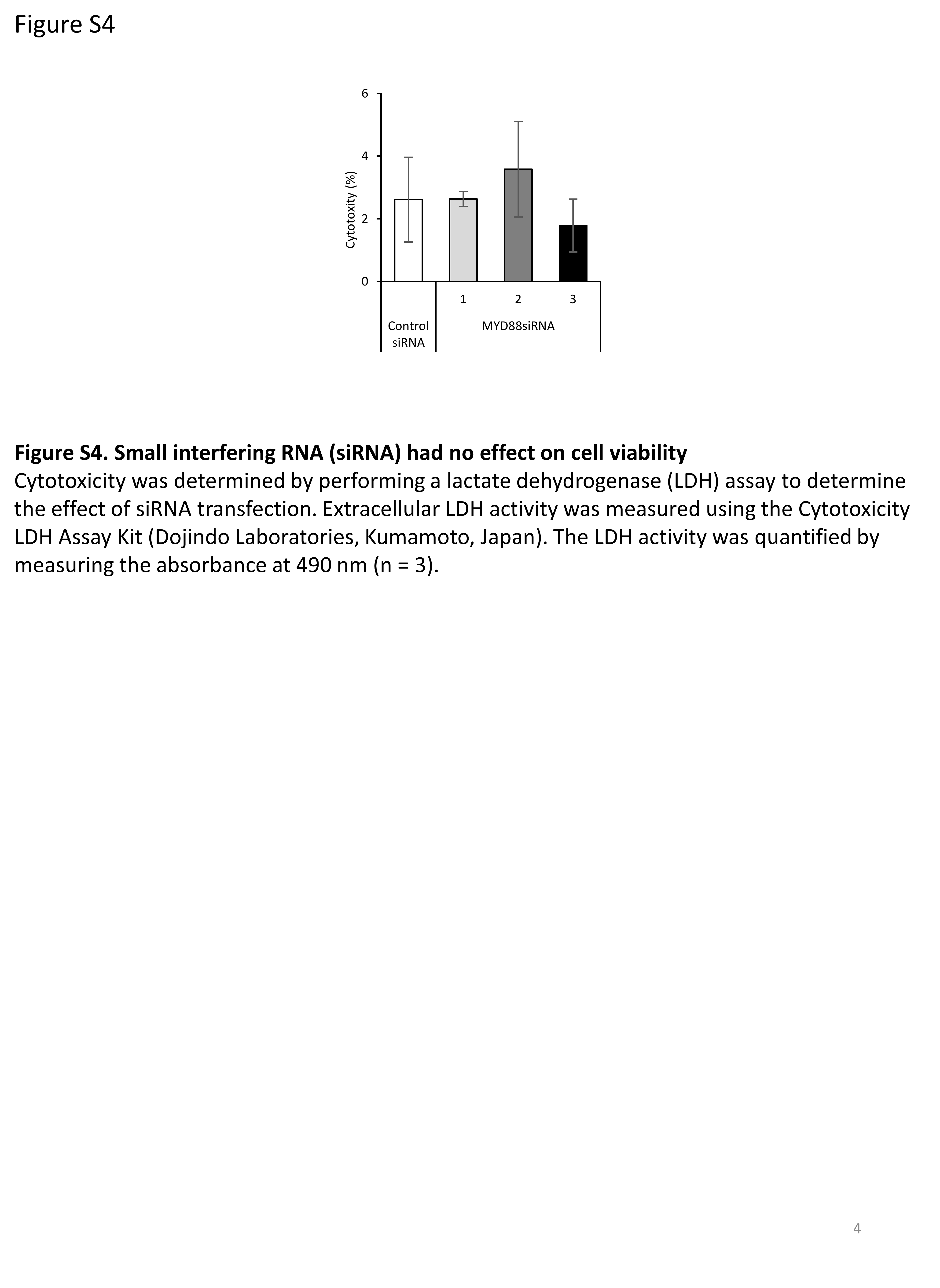

Supplement: Supplementary file 1 [file nutrients-15-02655-s001.zip › Figure S4.TIF]
